# Traffic-related air pollution and obesity formation in children: a longitudinal, multilevel analysis

**DOI:** 10.1186/1476-069X-13-49

**Published:** 2014-06-09

**Authors:** Michael Jerrett, Rob McConnell, Jennifer Wolch, Roger Chang, Claudia Lam, Genevieve Dunton, Frank Gilliland, Fred Lurmann, Talat Islam, Kiros Berhane

**Affiliations:** 1 Division of Environmental Health Sciences, School of Public Health, University of California, 50 University Hall MC7360, Berkeley, CA, USA; 2 Department of Preventive Medicine, Keck School of Medicine, University of Southern California, Los Angeles, CA, USA; 3 College of Environmental Design, University of California, Berkeley, CA, USA; 4 Sonoma Technology, Petaluma, CA, USA

**Keywords:** Childhood obesity, Air pollution, Traffic, California

## Abstract

**Background:**

Biologically plausible mechanisms link traffic-related air pollution to metabolic disorders and potentially to obesity. Here we sought to determine whether traffic density and traffic-related air pollution were positively associated with growth in body mass index (BMI = kg/m^2^) in children aged 5–11 years.

**Methods:**

Participants were drawn from a prospective cohort of children who lived in 13 communities across Southern California (N = 4550). Children were enrolled while attending kindergarten and first grade and followed for 4 years, with height and weight measured annually. Dispersion models were used to estimate exposure to traffic-related air pollution. Multilevel models were used to estimate and test traffic density and traffic pollution related to BMI growth. Data were collected between 2002–2010 and analyzed in 2011–12.

**Results:**

Traffic pollution was positively associated with growth in BMI and was robust to adjustment for many confounders. The effect size in the adjusted model indicated about a 13.6% increase in annual BMI growth when comparing the lowest to the highest tenth percentile of air pollution exposure, which resulted in an increase of nearly 0.4 BMI units on attained BMI at age 10. Traffic density also had a positive association with BMI growth, but this effect was less robust in multivariate models.

**Conclusions:**

Traffic pollution was positively associated with growth in BMI in children aged 5–11 years. Traffic pollution may be controlled via emission restrictions; changes in land use that promote jobs-housing balance and use of public transit and hence reduce vehicle miles traveled; promotion of zero emissions vehicles; transit and car-sharing programs; or by limiting high pollution traffic, such as diesel trucks, from residential areas or places where children play outdoors, such as schools and parks. These measures may have beneficial effects in terms of reduced obesity formation in children.

## Introduction

Childhood obesity has emerged as a major public health problem in the United States and elsewhere. Since the 1970s rates of overweight and obesity have more than doubled in the U.S. from about 15% of youth aged 2–19 years who were considered overweight or obese, to 32% in 2003–2006 [1,2]. Although the trend toward increasing obesity in the U.S. appears to have abated over the past ten years [3], the existing high prevalence remains a concern. Similar patterns of increasing childhood obesity prevalence have been reported in several other countries [4]. The increased prevalence of overweight and obesity in children has serious ramifications for future trends of metabolic disorders and disease, cardiovascular and pulmonary disease, gastrointestinal conditions, skeletal problems, cancer incidence, mortality, and psychosocial disorders [5-7]. While genetic and metabolic susceptibilities exist, the rapid rise in obesity prevalence implicates environmental factors as contributors to obesity development in children [8].

Growing evidence links the built environment to physical activity, dietary intake, and obesity [9]. Previous research has examined the impacts of land use patterns such as “urban sprawl” [10], local land use mixtures [11], and accessibility of neighborhood features that either promote or undermine health (e.g., exercise facilities or fast food outlets) [12,13]. Much of the existing evidence comes from cross-sectional studies [14], raising questions of reverse causality whereby individuals and families who would have otherwise stayed at a healthy weight locate in neighborhoods that support their already active lifestyles and nutritional food intake.

Recently, researchers examined longitudinally the role of traffic density around the homes of children. They found that higher levels of vehicular traffic were associated with higher attained body mass index (BMI measured as kg/m^2^) in children aged 10–18 [15]. Traffic is associated with several adverse exposures including increased accident danger and air pollution [16], suggesting different explanations for the positive association between traffic and attained BMI. Heightened traffic danger may discourage children from engaging in active transport by foot or bicycle for utilitarian purposes [17], and other things being equal, this would lower overall physical activity and could contribute to a positive energy balance.

Other research indicates that air pollution exposure, with traffic as a major source in many cities, may operate through inflammatory pathways to initiate metabolic processes contributing to diabetes formation [18,19]. These findings are supported by animal research showing that mice fed a fat chow diet and exposed to air pollution develop more visceral fat and insulin resistance than mice eating the same diet, but breathing purified air [20].

At this time, there are few epidemiological studies that have investigated specifically whether air pollution contributes to obesity formation in childhood, and only one study has examined traffic density effects on BMI growth [15]. A recent study suggested that early life exposure to polyaromatic hydrocarbon markers of ambient traffic-related pollution were associated with subsequent increased BMI and obesity at age seven [21]. Here we aim to assess the impact of traffic-related air pollution and traffic density near the home on the growth of BMI in a prospective cohort of children who were followed from age 5–11 in 13 Southern California communities. This paper seeks to expand on the earlier assessment of traffic as a risk factor by examining the specific pathway of air pollution exposure. In this context, the main aim of the study is to assess whether exposure to traffic and traffic-related air pollution relate to BMI growth in children.

## Methods

### Conceptual framework

**Figure 1:**
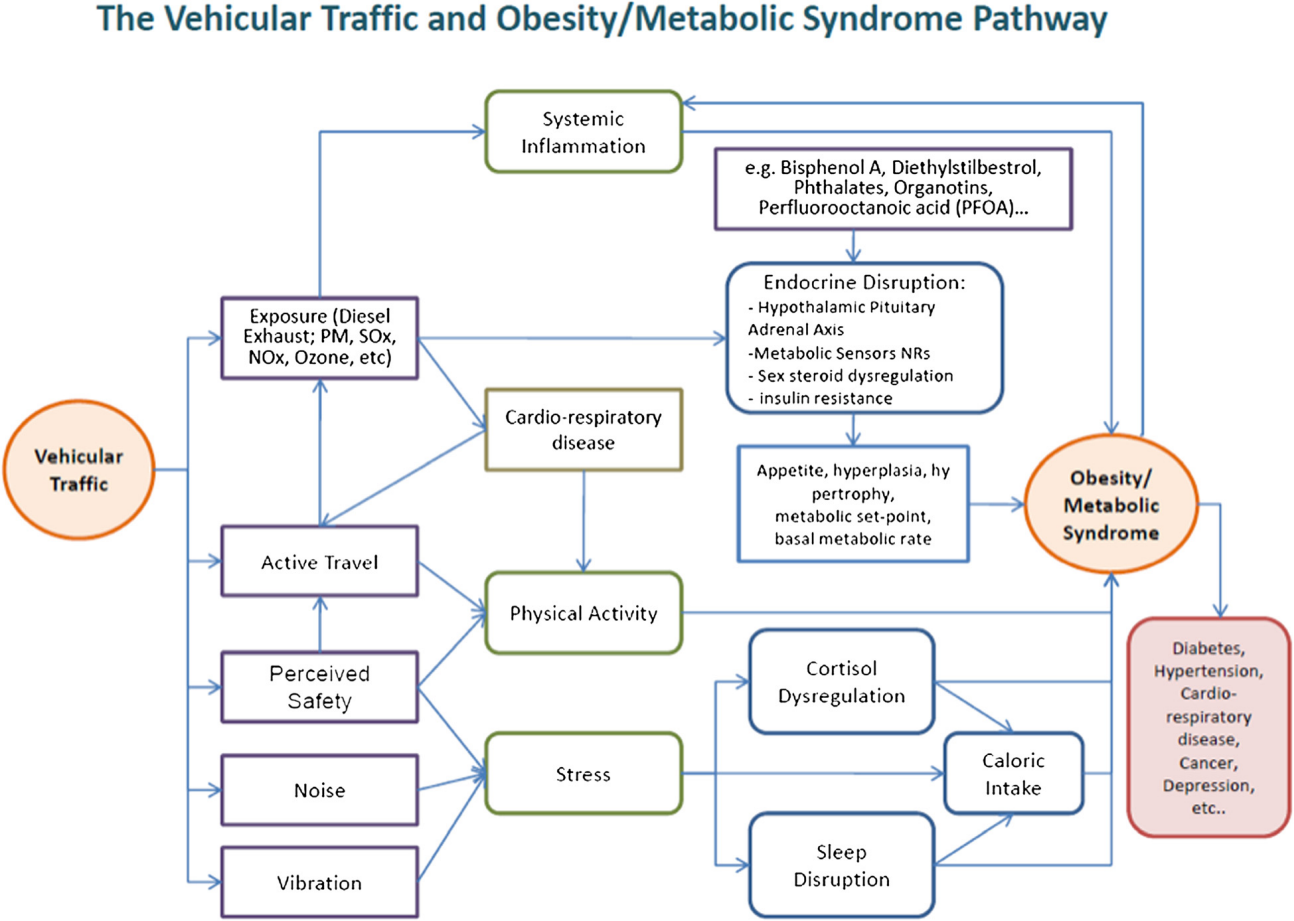
**Conceptual framework.** Conceptual framework illustrating pathways from vehicle traffic to obesity and metabolic syndromes.

In Figure 1, we propose the following conceptual model to illustrate the pathways through which traffic might affect obesity and cardio-metabolic disorders. Traffic could influence perceived safety and thereby affect the amount of active travel by foot or by bike. In this instance, we hypothesize that higher traffic could reduce physical activity, and as noted above, this could positively change energy balance. Previous research on this and similar cohorts has demonstrated that traffic can negatively affect active travel [22] and that this may lead to higher levels of obesity [15]. Another pathway could operate through perceived safety, noise and vibration, which all have the potential to increase stress. Stress has been associated with higher intakes of fat and carbohydrates and with cortisol and sleep dis-regulation that can affect the diet. All of these pathways, if they lead to altered eating habits, could contribute to obesity. In recent research on the same cohort, we showed that stress in the family is linked to small increases in BMI growth [23]. Finally, there is the impact of environmental and traffic-related pollution. Here the effect could operate through systemic inflammation to increase pro-obesogenic pathways mentioned above [20] or through the formation of chronic diseases that might lessen physical activity and have themselves been associated with obesity in the case of asthma [24,25]. Some components of traffic-related air pollution may contain endocrine disruptors that could be obesogens. This pathway might be enhanced through other obesogen exposures from other environmental sources such as phthalates [26]. This framework is used to guide our statistical modeling in terms of selecting variables to test for confounding and to help interpret our results where specific variables are unavailable for analyses (e.g., biomarkers of obesogen exposures).

### Ethics statement

The research protocol, including informed consent forms, was reviewed and approved by the Institutional Review Board, University of Southern California. Subsequent approval was given by the Committee on the Protection of Human Subjects, University of California, Berkeley for the geographic information exposure assignment to the homes of the study subjects.

### Study design

A cohort of children attending kindergarten and first grade (age 5–7 years) were enrolled during the 2002–03 school year from classrooms in 45 schools across 13 communities in Southern California (N = 4550). Parents provided informed consent and completed a detailed baseline and yearly follow-up questionnaires with information about asthma and related symptoms, demographic characteristics, physical activity, characteristics of homes, and other relevant covariates. Height and weight were measured without shoes at study entry and annually by a trained technician at the child’s school. Technicians followed a standardized procedure to measure height and weight using a calibrated medical scale. Measurements were recorded to the nearest 1 cm and 1 lb (0.45 kg), respectively. These objective measures of height and weight allowed for accurate calculation of BMI.

Other characteristics of this cohort have been described previously [27]. Information on demographic characteristics from questions that were asked repeatedly in yearly questionnaires was updated for this analysis. We also collected information on physical activity, mainly in the form of programmed activities and team sports (see Additional file 1 for details). The analytical data set was restricted to children who had two or more measurements of height and weight (N = 4257).

Homes of the children were geo-coded. Built-environment variables such as access to parks were calculated around the children’s homes and schools and assigned to each child (see [13,15] for more detail on the built environment variable compilation). Neighborhood and community social environmental variables, such as the poverty rate available from the U.S. Census, were also assigned to the residential address for inclusion as confounders in our multilevel models.

### Exposure models

Exposure to air pollution was assigned using the CALINE4 dispersion model (see Additional file 1 for details). Briefly, this model used Gaussian plume dispersion parameters with traffic data, emissions factors, and local meteorology to estimate exposure to the mixture of near-roadway pollutants at the homes of the children based on a model for the incremental increase in nitrogen oxides (NO_x_) above regional background levels, as previously described [27]. Exposures for freeway and non-freeway sources were assigned to the baseline address of the children.

Traffic exposure variables were based on the California Department of Transportation Functional Class (FC) data for the year 2000. The annual average daily traffic (AADT) volumes were conflated to the TeleAtlas road network [28]. Traffic data were based on continuous measurements on freeways, highways, and some major arterials, and intermittent measurements within the previous three years on other major roads. The spatial pattern of traffic density changes slowly over time and the temporal period used here likely supplies a good representation of the longer-term traffic patterns around the subjects’ homes for our study period. As described elsewhere, a kernel density function was estimated to smooth the influence of traffic around the home [15]. This function down-weighted the influence of traffic exposures as a function of Euclidian distance away from the child’s home. Based on previous evidence [15], traffic density was examined within 150 m of the home.

### Statistical methods

A multilevel linear model was used that allowed for examination of the effects that risk factors have on attained BMI level at age 10 and the rate of growth during the follow-up period between the ages of 5–11 years [29,30]. This modeling approach properly adjusts for age- and sex- specific effects on BMI growth in children, provides an effective mechanism for assessing effects of risk factors on BMI level and growth, and also implicitly adjusts for baseline levels of BMI. Letting *c, i* and *j* denote the study community, child and year of measurement, respectively, the following multi-level linear model was used to examine the effect of an exposure variable (e.g., NOx) at the individual level, *X*_*ci*_, on BMI, *Y*_*cij*_:

(1) $$\mathrm{Level}\;1:\;Y_{\mathrm{cij}}=A_{\mathrm{ci}}+B_{\mathrm{ci}}t_{\mathrm{cij}}+e_{\mathrm{cij}}$$

(2) $$\mathrm{Level}\;2a:\;A_{\mathrm{ci}}=A_{c}+\beta_{1}X_{\mathrm{ci}}+\delta_{1}Z_{\mathrm{ci}1}+\cdots +\delta_{q}Z_{\mathrm{ciq}}+e_{\mathrm{ci}}$$

(3) $$\mathrm{Level}\;2b:\;B_{\mathrm{ci}}=\beta_{0}+\beta_{2}X_{\mathrm{ci}}+f_{\mathrm{ci}}$$

where *t*_*cij*_ denotes age of participants at time of BMI measurements (centered at 10 years of age), *A*_*c*_ denotes town specific intercepts, and *Z*_1_, …, *Z*_*q*_ denote adjustment factors such as sex, and race/ethnicity categories. Our results were obtained by combining equations (1–3) to fit the following unified mixed effects model:

(4) $$\begin{array}{ll} Y_{\mathrm{cij}}= & A_{c}+\beta_{0}t_{\mathrm{cij}}+\beta_{1}S_{\mathrm{ci}}+\beta_{2}S_{\mathrm{ci}}\times t_{\mathrm{cij}}+\delta_{1}Z_{\mathrm{ci}1}\\ & +\cdots +\delta_{q}Z_{\mathrm{ciq}}+e_{\mathrm{ci}}+f_{\mathrm{ci}}t_{\mathrm{cij}}+e_{\mathrm{cij}} \end{array}$$

In Eqn (4), *β*_1_ and *β*_2_ correspond to the simultaneously estimated effects of exposure on BMI level attained at age 10 (i.e., examining the main effect between individuals) and also the yearly slope of change in BMI during the follow-up period, respectively. Random effects for community were used in models that assessed confounding by community level covariates such as poverty and crime rates, essentially leading to three-level models.

This modeling approach allowed for examination of the effects of covariates of interest at various levels: between times (within individual), between individuals, and between other levels of spatial aggregation (e.g., school or community). The base model included indicator functions for community, gender, and race or ethnicity. A final model was then developed by including all additional confounders that individually changed the effect of interest on the attained BMI level at age 10 (level) or the rate of change in BMI levels (slope) by at least 10%. All confounders were included for both “level” and “slope”. Analyses were conducted using SAS (Cary, NC, U.S.) and R (Vienna, Austria) statistical software packages.

In these multilevel models, more than 50 confounding variables were screened at the individual, neighborhood, school, and community scales. As a sensitivity analysis, models with both random and fixed effects clustered on the schools of the children were also run.

## Results

**Figure 2:**
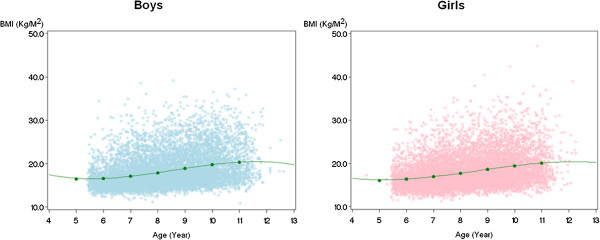
**BMI growth curves for boys and girls over the follow-up.** Points show individual BMI measures for the subjects.

The mean age of children at study entry was 6.6 years (standard deviation (SD) 0.65; range 4.5-8.9). Average BMI was 16.79 at study entry (SD 2.81) (Table 1). By year 5 of the study BMI had increased approximately 2.6 units to 19.35 (SD 4.21) with boys showing a slightly greater increase. Based on Centers for Disease Control percentiles between the 85th and 95th percentile, rates of overweight were 14.4%. Obesity rates measured as BMI scores equal to or greater than the 95th percentile were 15%. The growth curves for BMI in boys and girls are shown in Figure 2. The slope of the growth curve over the follow-up period did not deviate from a linear trend.

**Table 1:** **Participant baseline**^**a **^**characteristics, exposures and potentially confounding variables used in the analysis**

**Participant characteristics**	**No.**	**(%)**	**Mean**	**(SD)**
Race/Ethnicity				
African American	122	(2.68)		
Asian	145	(3.19)		
Hispanic	2462	(54.11)		
Non-Hispanic White	1664	(32.18)		
Other	357	(7.85)		
Gender				
Male	2297	(50.51)		
Female	2251	(49.49)		
**Individual and household characteristics**				
Parental Education				
Less than High School	905	(21.75)		
High School	781	(18.77)		
Above High School	2575	(59.48)		
Second hand smoke				
No one ever smoked in the house	3962	(97.22)		
Anyone ever smoked in the house	309	(7.23)		
Ever Asthma				
No	3501	(86.13)		
Yes	564	(13.87)		
Spanish Speaker				
No	3417	(75.1)		
Yes	1133	(24.9)		
**Local home or school environment**				
Having no food stores within 500 m road network buffer				
No	1980	(48.09)		
Yes	2137	(51.91)		
Street connectivity				
(Gamma index 500 m buffer)	4117		0.4	0.06
Parks and recreation				
(unit: acre in 500 m buffer)	3968		4.95	10.6
NDVI green cover^b^ (in 500 m buffer)	4117		0.09	0.10
Recreation programs within 5 km	4117		29.7	34.20
**Community social context**				
Proportion of unemployed males and females			0.076	0.02
Community level violent crime rate				
(Crimes per 100,000 population)	4550		511.73	268.04
**Air pollution and traffic**				
Total NO_X_ (parts per billion)	4464		49.24	104.93
Traffic density within 150 m of the home	4464		19.49	18.82
**Primary outcome**				
BMI at baseline	4550		16.79	2.81
Males	2297		16.87	2.81
Females	2251		16.70	2.80
BMI at the end of follow up	4550		19.35	4.21
Males	2297		19.50	4.36
Females	2251		19.19	4.15
BMI CDC percentile at baseline				
85 > BMIp	3201	(70.35)		
85 ≤ BMIp < 95	660	(14.41)		
95 ≤ BMIp	684	(15.03)		

^a^First observation of the subjects in the first year of the study is N = 4550 with restriction of non-missing BMI and with two or more observations; numbers of subjects in the table vary due to missing covariate values.

^b^Normalized difference vegetation index derived from Landsat satellite images.

Traffic density at 150 m radius had a positive, but borderline significant (p <0.1) association with the intercept and the slope of BMI growth curves of the children (Table 2). Further evaluation of the traffic effects revealed that they were confounded by other variables, particularly whether the questionnaire had been completed in Spanish, suggesting the child was from a family of recent immigrants from Latin America. A final model included asthma status of the child, the language used to complete the questionnaire (Spanish or English), whether the child was exposed to second-hand smoke in the home, the parental level of education, the gamma index (a measure of the connectivity of the street network around the child’s home which affects walking distances), the number of fast food outlets within 500 m of the child’s home, greenness around the home as measured by the normalized difference vegetation index, the number of active recreational programs for children offered within 5 km of the home, and traffic density at 150 m (Table 2). In this fully-adjusted model, the effect of traffic within 150 m remained positive on the slope, but was reduced by more than 20% by the confounders and was no longer borderline significant. Of note, we tested several variables measuring various aspects of physical activity or participation in sports, but none of these variables met our inclusion criteria for confounding.

**Table 2:** Effects of traffic density or traffic-related air pollution on BMI level (intercept) and growth (slope)

	**Combined effect modeling**	
Exposure based on 10-90th percentile contrast	Male and Female	
	Intercept β (SE)	Slope β (SE)
Traffic density^a^	0.0012* (0.0006)	0.0002* (0.0001)
Non-Freeway NO_x_^a^	0.3831** (0.1552)	0.0861** (0.0255)
Traffic density^b^	0.0012* (0.0006)	0.0002 (0.0001)
Non-Freeway NO_x_^b^	0.3867** (0.1552)	0.0873** (0.0255)

**p < 0.05.

*p < 0.1.

^a^Models include the same confounders: whether the child has ever had asthma, parental education as a marker for socioeconomic position, whether the questionnaire was answered in Spanish as a marker for recent immigrant status, normalized difference vegetation index within 500 m of the home as a measure of green cover, street connectivity as measured by the gamma index, recreational programming within 5 km of the home, and fast food access within 500 m of the home.

^b^Confounders selected based on modeling procedure described in the methods for each exposure. The traffic density model includes parental education as a marker for socioeconomic position, whether the questionnaire was answered in Spanish as a marker for recent immigrant status, normalized difference vegetation index within 500 m of the home as a measure of green cover, and recreational programming within 5 km of the home.

The non-freeway NO_x_ model includes parental education as a marker for socioeconomic position, whether the questionnaire was answered in Spanish as a marker for recent immigrant status, normalized difference vegetation index within 500 m of the home as a measure of green cover, street connectivity as measured by the gamma index, recreational programming within 5 km of the home, and fast food access within 500 m of the home.

All of the above models include indicator functions for community of residence and variables for sex and race or ethnicity.

In the screening of the air pollution variables, non-freeway NO_x_ levels were significantly and positively associated with BMI at age 10 and the rate of growth over the four year follow-up period, while the freeway-related exposures were not associated with BMI growth, consistent with other previous studies on respiratory health [27]. The association between BMI and non-freeway NO_x_ was reduced but remained significantly elevated in models containing the same variables as those in the fully adjusted traffic density model described above and with those chosen specifically to confound NO_x_ (Table 2). Again none of the physical activity variables met the inclusion criteria as confounders. Interaction by gender was tested, but no significant evidence of difference in the effects on boys and girls was found.

Confounders at the community and school levels were further tested by including the average terms for each level and the individual deviations from the mean of the level. Neither community level crime nor poverty confounded the within-community effect of air pollution. The impact of the school level was then tested by including a fixed effect for school in the model, but air pollution remained significantly and positively associated with BMI growth with little change in the coefficient. This suggests that the school level variables did not confound the air pollution effect on BMI growth.

## Discussion

We hypothesized that traffic density and traffic-related air pollution would positively associate with longitudinal growth in BMI. In this cohort of children from 13 communities across Southern California, traffic-related air pollution exerted a significant effect on BMI growth and BMI level attained at age 10. Evidence of effects for traffic density was found in unadjusted models. This effect was confounded in fully adjusted models, although the effects did remain elevated.

**Figure 3:**
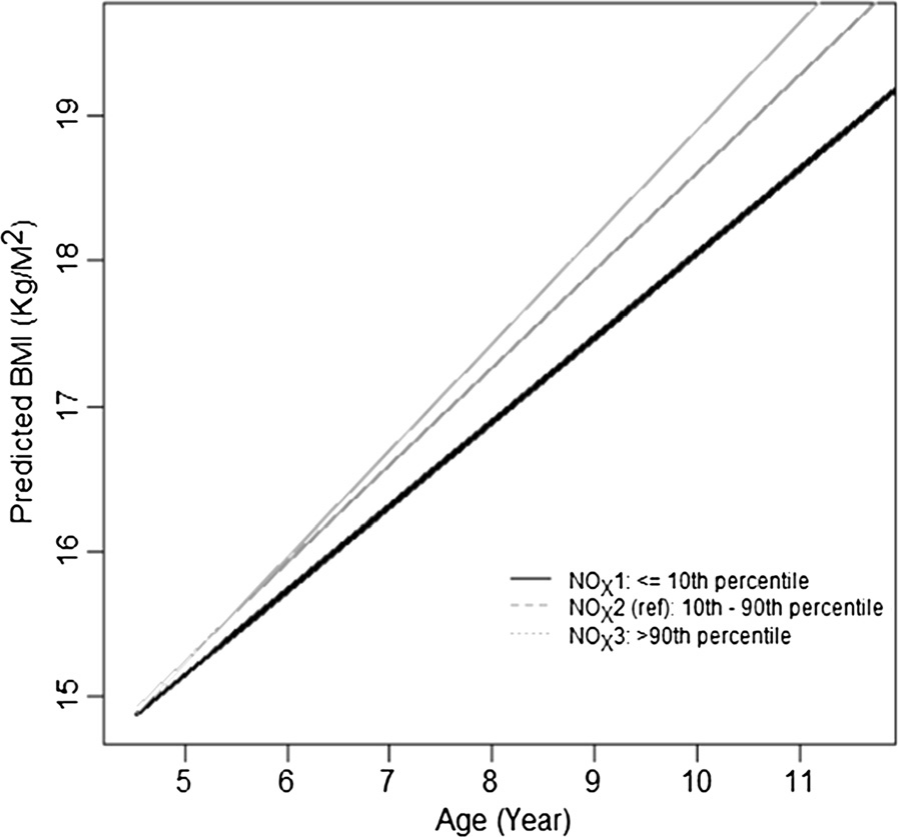
**Predicted BMI.** Plot of predicted BMI comparing children in the 10th and the 90th percentiles with the 10-90th percentile exposure contrast shown for reference.

Comparing children in the highest 10% of traffic-related air pollution exposure to those in the lowest 10% of exposure yielded a 0.39 BMI unit increase in the attained BMI level at age 10. This translated into a 13.6% increase in the rate of average annual BMI growth. These effects may have large population impacts because traffic-related air pollution is a ubiquitous exposure that affects billions of people globally [31], and in many countries traffic is increasing at a higher rate than the rate of population growth [32].Examining the effects at different times during the follow up helps to interpret the results. Figure 3 shows the BMI effects for the children in the lowest and highest deciles. The BMI range between the lowest and highest is shown as the middle line for reference. As the children get older, the effects accumulate, and the slope difference between the lowest and highest deciles becomes more pronounced. By age 10 or 11 the difference is about 0.4 – 0.5 of a BMI unit.

Traffic density and traffic-related air pollution could not be tested in the same model because traffic density is an input variable to the dispersion models, and therefore the two variables are collinear. Traffic-related air pollution nonetheless was not confounded by other variables, suggesting that air pollution exerted a stronger effect on BMI growth than traffic density. This result was insensitive to which individual and neighborhood built environment confounding variables were used in the model. Based on the sensitivity analyses, variables at the school and community level do not confound the association between BMI and traffic-related air pollution.

The findings here differ from the only other study that examined the impacts of traffic density on BMI growth [15], which was conducted among an older cohort of children in 10 of the same study communities and used similar statistical techniques. With the same metric of traffic density within 150 m around the home, the earlier study found significant effects that were not confounded by other individual or built environment variables or community-level variables such as poverty. This difference in findings from the two cohorts may have resulted from mobility differences by age. Most of the children in the present analysis were less than 10 years old for most of the follow up, and children of this age are less likely to walk on their own than older children who were followed for the earlier research [15]. Qualitative research suggests that parents of children aged less than 10–11 perceive many barriers to allowing children to move freely in urban areas, but the same study indicates that at this age, which corresponds to the end of primary school, parents do begin to afford increased license to engage in physical activity alone or more likely in groups of peers [33]. Quantitative research using global positioning systems to track children supports the qualitative research, indicating that there is a large rise in the proportion of children allowed to range freely around the ages of 10–11 [34]. Therefore, the pathway of reduced physical activity from traffic danger in younger children may be less pronounced in older children, because fewer of the younger children exhibited independent mobility on average. The earlier study on traffic density did not test for associations with traffic-related air pollution.

Reliance on the CALINE4 dispersion model limited our ability to discern which elements of the traffic pollution mixture were most important. Although we used NOx as our indicator of traffic-related pollution, this molecular gas had strong correlations with CO, NO_2_, and PM_2.5_ estimates from the CALINE4 model, with correlations greater than 0.9 (see Additional file 1 for further details). We found non-freeway NOx had the association, while freeway NOx was not robust to confounders. We interpret the lack of effect from the freeway NOx as resulting from a small proportion of the total cohort who lived in proximity to freeways, rather than an attribution to a specific source from a different type of roadway. While the results indicate that traffic-related pollution likely has a stronger effect than traffic density, we are unable to identify which specific components of the traffic mixture were responsible for the effects.

Another limitation of this study related to the lack of information on food intake. Food access was controlled in the models, but dietary factors could not be directly evaluated. Given what is known about the association between lower socioeconomic position and higher traffic-related pollution exposures in California [35], some of the effects observed here may be confounded by dietary variables that are also associated with lower socioeconomic status, such as intake of sugar and fats [36]. Socioeconomic status in the home and neighborhood was controlled for, which reduces the chance of residual confounding relating to socioeconomic status, but confounding by food intake, which might be associated with air pollution through socioeconomic status, cannot be directly ruled out.

To address the concern about diet, information on dietary intake in an older cohort (ages 10–18) of nearly 2000 children in 10 of the same study communities as in the current study [37,38] was used to generate variables on macronutrients including total caloric, protein, carbohydrate, saturated fat, mono unsaturated fat, and cholesterol intake. A statistical analysis that controlled for community of residence, race, sex, and parental education as a marker of SES was performed, and for a wide array of traffic or traffic-pollution indicators there was no association between the total caloric intake and the traffic-pollution estimates or traffic density measures. A weak, borderline significant association between daily grams of carbohydrate consumption and nitrogen dioxide from non-freeway sources was observed, but the coefficient was very small. Equivalent diet information on the specific cohort used in our paper is not available, but the relationships between the traffic or pollution variables and food intake should be similar in both cohorts. Given that there was no difference in total calories or in any other macronutrient categories, the chance of confounding by unmeasured diet variables is limited.

Although we cannot rule out self selection of potentially more health-conscience families into areas with lower pollution, our mixed effects modeling framework properly controls for baseline BMI. As a result, the influence of self-selection is accounted for with subject-to-subject variability due to baseline characteristics. While self-selection could influence the trajectory, control for baseline characteristics that is inherent to our modeling framework makes it more likely that our results are from an ongoing influence of the environment and not some other factors.

The effects of pollution are significant, and the temporal pattern is consistent with the hypothesis that the inflammatory effects of air pollution predispose children to obesity in a similar way to what has been observed in laboratory experiments [20]. By analogy, this pattern is also corroborated by human epidemiological studies finding associations between metabolic disorders and air pollution [18,19]. Another explanation is possible; in areas of high traffic, children and their parents may have a heightened sense of danger that reduces activity by restricting the mobility of families [39]. In this cohort, however, traffic effects were not significantly associated with BMI growth or attained level after controlling for confounding variables. As illustrated in our conceptual framework presented in Figure 1, there are several other pathways from stress resulting from noise or from other obesogens, which could be leading to higher BMI growth in children, but we are unable to test such pathways directly. Future research may usefully address these other pathways along with traffic pollution exposures.

## Conclusions

This paper provides evidence that traffic-related air pollution is associated with the development of obesity in children. Traffic pollution may be controlled via emission restrictions; changes in land use that promote jobs-housing balance and use of public transit and hence reduced vehicle miles traveled; promotion of zero emissions vehicles; transit and car-sharing programs; or by limiting high pollution traffic, such as diesel trucks, from residential areas or places where children play outdoors, such as schools and parks. These measures may have beneficial effects in terms of reduced obesity formation in children.

## Competing interests

Dr. McConnell has received research support from an air quality violations settlement between the South Coast Air Quality Management District, a California state regulatory agency, and BP. The authors have no other conflicts of interest to disclose.

## Authors’ contributions

Conceived and designed the experiments: MJ RM JW KB. Performed the experiments: RM RC CL FL. Analyzed the data: MJ RM JW RC CL GD FG. Wrote the first draft of the manuscript: MJ RM JW GD FL KB. Contributed to the writing of the manuscript: MJ RM JW RC CL GD FG FL KB. ICMJE criteria for authorship read and met: MJ RM JW RC CL GD FG FL KB. Agree with manuscript results and conclusions: MJ RM JW RC CL GD FG FL KB. All authors read and approved the final manuscript.

## Supplementary Material

Additional file 1 Additional information on physical activity and exposure assessment. Click here for file
