# Supplementary material for: Traffic-related air pollution and obesity formation in children: a longitudinal, multilevel analysis
Source: Environ Health. 2014 Jun 9;13:49. doi: 10.1186/1476-069X-13-49 (PMC4106205; doi:10.1186/1476-069X-13-49)
Supplement: Additional file 1 — Additional information on physical activity and exposure assessment. [file 1476-069X-13-49-S1.pdf]

Additional File 1

Information on Physical Activity Assessment

Parents reported information about their child's participation in outdoor organized team sports at baseline. We adapted from the M-SPAN intervention evaluation instrument [1], which has been shown to have acceptable levels of reliability. Parents were asked the following: "*Since September of this school year, has your child played outdoors in any organized team sports at least twice per week*?" Examples included baseball/softball/t-ball, soccer, swimming, basketball, football, tennis volleyball, skating/roller blading, track/field, golf, other. We dichotomized responses as "*Yes- participated in outdoor organized team sports at least twice per week*" and "*No-did not participate in outdoor organized teams sports at least twice per week.*" We also collected information about participation in non-school indoor structured activity programs through the following question, "*Has your child taken any of the following exercise classes, lessons, or special programs during that past 12 months (outside of school only)?*" Response options included dance, aerobics, gymnastics or tumbling, martial arts, other, and none of the above. Parents were instructed to mark all that apply. Responses were recoded as *0 programs, 1 program, or 2+ programs* (treated ordinally) for our analyses.

Near-roadway air pollution exposure methods

This Appendix is adapted from McConnell et al. 2010 [2]. Participant residence and school addresses were standardized and their locations were geo-coded to 13 m perpendicular to the side of the adjacent road, using the Tele Atlas database and software (Tele Atlas, Inc., Boston, CA, www.na.teleatlas.com). Distance to the nearest major road was estimated using ESRI ArcGIS Version 8.3 (ESRI, Redlands, CA, www.esri.com). We included in the analysis only children with addresses that could be geo-coded accurately. Specifically, only residential addresses for which the TeleAtlas geo-coding software assigned its highest quality match code were included. These addresses were located on the correct side of the street with their relative position between cross streets determined by linear interpolation of residence number between the nearest intersections.

Annual average daily traffic volumes on roadways were obtained from the California Department of Transportation Highway Performance Monitoring System (CalTrans 2002). Using previously described methods, the traffic volumes were transferred from the Department of Transportation roadway network to the TeleAtlas networks [3]. Annual average exposure to local traffic-related pollutants at homes and schools was estimated from CALINE4 dispersion models that incorporate distance to roadways, vehicle counts, vehicle emission rates, wind speed and direction, and height of the mixing layer in each community [4]. Separate estimates were made for the contribution of local traffic on freeways and on all other roadways to concentrations of several pollutants, including carbon monoxide, nitrogen dioxide,

total oxides of nitrogen, elemental and organic carbon and PM_{10} and $PM_{2.5}$. These estimated pollutant exposures should be regarded as indicators of incremental increases due to primary emissions from local vehicular traffic on top of background ambient levels, based on annual average estimates. Modeled total oxides of nitrogen (NO_x), for example, which we used to evaluate associations with BMI, represented only the effect of the incremental contribution of local traffic to a more homogeneous community background concentration of NO_x that included both primary and secondary pollution resulting from long range transport and regional atmospheric photochemistry. This metric was highly correlated with other pollutants estimated by CALINE4 ($R>0.95$). Therefore, modeled NO_x represented primary local NO_x from vehicular traffic, these other highly correlated traffic-related air pollutants, and probably other pollutants for which we did not estimate exposures.

References:

1. McKenzie TL, Sallis JF, Prochaska JJ, Conway TL, Marshall SJ, Rosengard P: **Evaluation of a two-year middle-school physical education intervention: M-SPAN.** *Med Sci Sports Exerc* 2004, **36**:1382–8.

2. McConnell R, Islam T, Shankardass K, Jerrett M, Lurmann F, Gilliland F, Gauderman J, Avol E, Künzli N, Yao L, Peters J, Berhane K: **Childhood incident asthma and traffic-related air pollution at home and school.** *Environ Health Perspect* 2010, **118**:1021–6.

3. Wu J, Funk TH, Lurmann FW, Winer AM: **Improving Spatial Accuracy of Roadway Networks and Geocoded Addresses**. *Trans GIS* 2005, **9**:585–601.

4. Benson PE: *CALINE-4: A Dispersion Model for Predicting Air Pollutant Concentrations Near Roadways, Report No. FHWA/CA/TL-84/15*. Sacramento, CA; 1989.

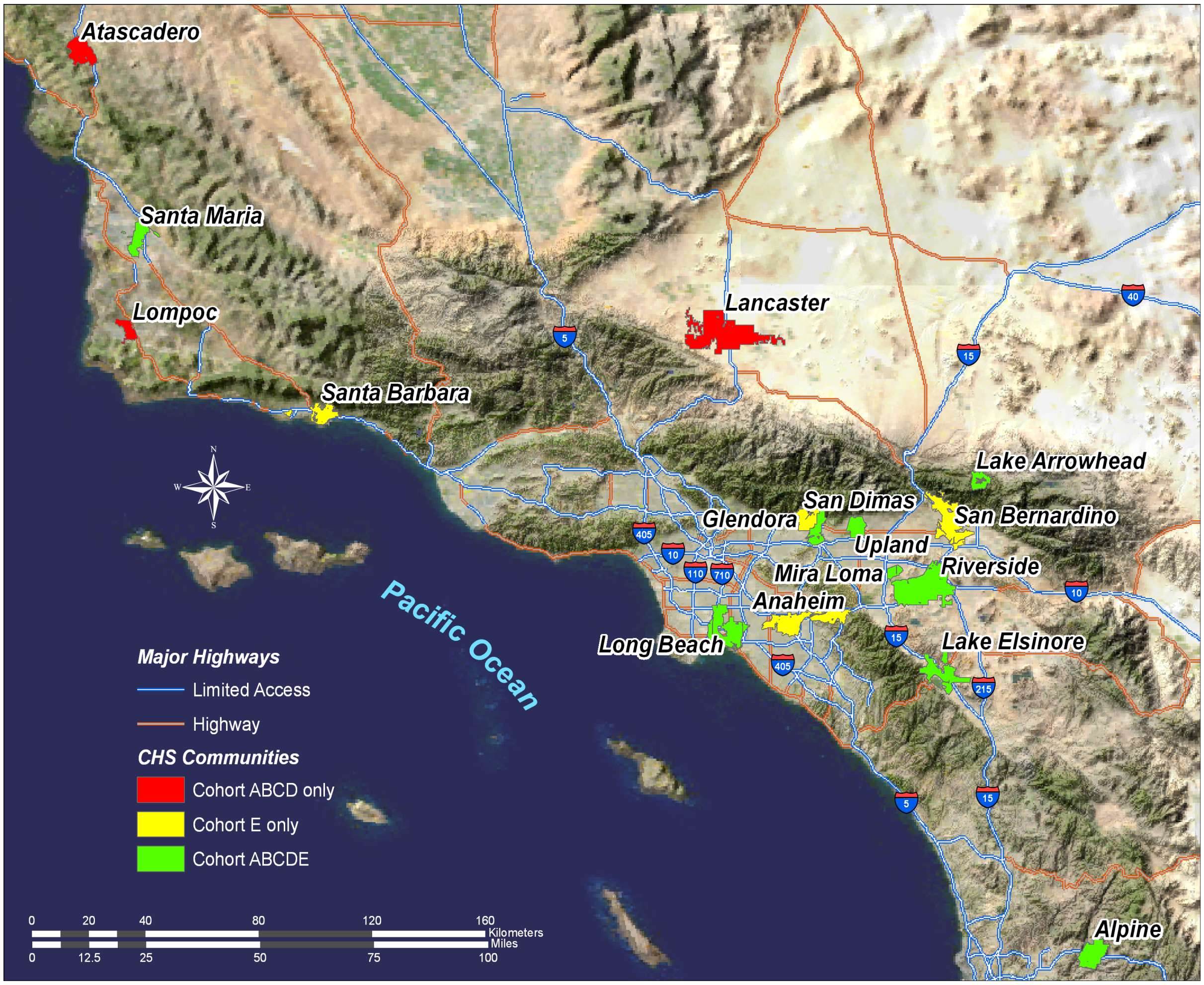

Figure A1. Map of the Study Area with Communities Show. Cohort E in yellow are the subjects used in this study; Cohort ABCD in red is the older cohort used for the nutritional analysis. Spatial overlap between the two cohorts is shown in green.
